# A standardised protocol for texture feature analysis of endoscopic images in gynaecological cancer

**DOI:** 10.1186/1475-925X-6-44

**Published:** 2007-11-29

**Authors:** Marios S Neofytou, Vasilis Tanos, Marios S Pattichis, Constantinos S Pattichis, Efthyvoulos C Kyriacou, Dimitris D Koutsouris

**Affiliations:** 1 Department of Computer Science, University of Cyprus (UCY), Nicosia, Cyprus; 2 Aretaeion Medical Center, Nicosia, Cyprus; 3 Department of Electrical and Computer Engineering, University of New Mexico, NM, USA; 4 Biomedical Engineering Laboratory, Division of Information Transmission Systems and Material Technology, School of Electrical and Computer Engineering, National Technical University of Athens (NTUA), Athens, Greece

## Abstract

**Background:**

In the development of tissue classification methods, classifiers rely on significant differences between texture features extracted from normal and abnormal regions. Yet, significant differences can arise due to variations in the image acquisition method. For endoscopic imaging of the endometrium, we propose a standardized image acquisition protocol to eliminate significant statistical differences due to variations in: (i) the distance from the tissue (panoramic vs close up), (ii) difference in viewing angles and (iii) color correction.

**Methods:**

We investigate texture feature variability for a variety of targets encountered in clinical endoscopy. All images were captured at clinically optimum illumination and focus using 720 × 576 pixels and 24 bits color for: (i) a variety of testing targets from a color palette with a known color distribution, (ii) different viewing angles, (iv) two different distances from a calf endometrial and from a chicken cavity. Also, human images from the endometrium were captured and analysed. For texture feature analysis, three different sets were considered: (i) Statistical Features (SF), (ii) Spatial Gray Level Dependence Matrices (SGLDM), and (iii) Gray Level Difference Statistics (GLDS). All images were gamma corrected and the extracted texture feature values were compared against the texture feature values extracted from the uncorrected images. Statistical tests were applied to compare images from different viewing conditions so as to determine any significant differences.

**Results:**

For the proposed acquisition procedure, results indicate that there is no significant difference in texture features between the panoramic and close up views and between angles. For a calibrated target image, gamma correction provided an acquired image that was a significantly better approximation to the original target image. In turn, this implies that the texture features extracted from the corrected images provided for better approximations to the original images. Within the proposed protocol, for human ROIs, we have found that there is a large number of texture features that showed significant differences between normal and abnormal endometrium.

**Conclusion:**

This study provides a standardized protocol for avoiding any significant texture feature differences that may arise due to variability in the acquisition procedure or the lack of color correction. After applying the protocol, we have found that significant differences in texture features will only be due to the fact that the features were extracted from different types of tissue (normal vs abnormal).

## Background

In the United States, in 2007, it is estimated that over 39,080 new cases will be diagnosed with gynaecological cancer of the endometrium, with an estimated 7,400 deaths [1]. Within the female population, gynaecological cancer accounts for the second highest mortality rate. Early diagnosis and treatment of gynaecological cancer are essential for better quality of life and longer life.

The development of minimally invasive surgery has presented the possibility of new approaches to certain longstanding problems in gyneacology. The initial efforts with hysteroscopy, transabdominal/transvaginal laparoscopy operations have already demonstrated the advantages of endoscopic techniques over traditional open and endovascular approaches. The advantages of laparoscopic/hysteroscopic methods are especially significant in patients with a low risk factor when the operation is usually prophylactic [2].

In laparoscopic/hysteroscopic imaging, the physician guides the telescope inside the uterine or abdominal cavity investigating the internal anatomy, in search of suspicious, cancerous lesions [3]. During the exam, the experience of the physician plays a significant role in identifying suspicious regions of interest (ROIs), where in some cases, important ROIs might be ignored and crucial information neglected [4]. The analysis of endoscopic imaging is usually carried out visually and qualitatively [5], based on the subjective expertise of the endoscopist. Therefore, this procedure suffers from interpretational variability, lack of comparative analysis and it is time consuming.

The objective of this study is to propose a standardized protocol for eliminating significant differences in texture feature analysis of endoscopy images. For gynaecological cancer, we show that the proposed approach eliminates significant statistical differences due to variations in: (i) the distance from the tissue (panoramic vs close up), (ii) difference in viewing angles and (iii) color correction. We validate the approach for texture features extracted at difference viewing conditions from: (i) calf endometrium chosen for its resemblance to human tissue, (ii) chicken cavities chosen for providing a more realistic laparoscopy/hysterocscopy operation environment, and then verify the findings for (iii) human subjects.

To the best of our knowledge, there are no other studies proposing a standardized quantitative image processing and analysis procedure for the laparoscopic/hysteroscopic imaging for gynaecological cancer. Several endoscopic studies have been reported related to standardisation, that focused on comparing treatment methods (not image processing standardization methods) and extracting conclusions about the performance and diagnosis for the endometrium [3]. On the other hand, several CAD systems have been reported for colonoscopy with highly promising results [6,7].

In this paper a standardized procedure based on color imaging correction and texture feature extraction and analysis is investigated for the analysis of gynaecological tissue. The gamma correction algorithm which is used extensively in many applications for correcting the camera images is applied for correcting the endoscopy images [8]. The usefulness of gamma correction was also demonstrated on endoscopic videos [9]. Applying gamma correction on the images, will also limit the variability when analyzing images captured with different cameras, telescopes and endoscopic hardware.

We investigate the use of texture features extracted from Regions of Interest (ROIs) from different types of tissue [10]. Textural information has been used extensively for the characterization of various tissues in endoscopic imaging, such as in colonoscopy [11-13], laryngoscopy, [14] and others. Several textural features were computed in this work based on Statistical Features (SF) [15], Spatial Gray Level Dependence Matrices (SGLDM) [16] and Gray level difference statistics (GLDS) [17].

In what follows, we provide details on the methodology, the results, discussion and concluding remarks.

## Methods

**Figure 1:**
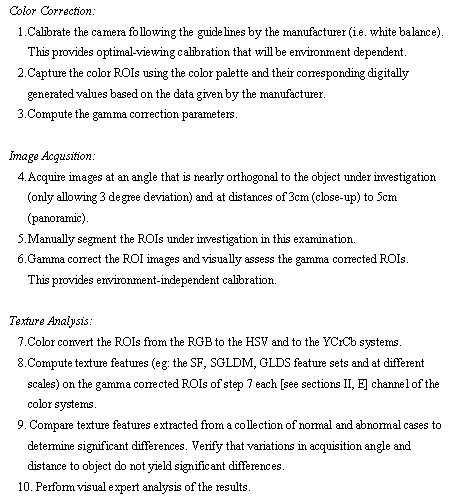
Image acquisition and analysis protocol.

We summarize the proposed protocol in Figure 1. The proposed approach is summarized in three parts. First, we perform color correction to compensate for lighting variations. Second, we acquire clinical images while carefully controlling the angle and distance to the subject. Third, we perform texture analysis through statistical analysis of the extracted texture features.

In the rest of the methods section, we provide details of the video acquisition, the color correction and the texture feature extraction. We also provide detailed descriptions of the clinical datasets. We provide a statistical analysis in the results section.

### Recording of endoscopic video

For image acquisition, we used the medical telescope provided by Wolf [22]. The telescope specifications were: 2,8 mm diameter and 30 degrees viewing angle. Endoscopy video was captured using the Circon IP4.1 RGB video camera [23]. All videos were captured at clinically optimum illumination and focusing. The camera was white balanced using a white surface (white color of the palette) as suggested by the manufacturer. The light source was a 300 Watt Xenon Light Source from ACMI Corporation [23]. The analog output signal from the camera (PAL 475 horizontal lines) was digitized at 720 × 576 pixels using 24 bits color and 25 frames per second at a resolution of approximately 15 pixels/mm, for the panoramic view and at approximately 21 pixels/mm for the close up view. The video was saved in AVI format. Digitization was carried out using the Digital Video Creator 120 frame grabber [24] that was connected to the PC through the IEEE 1394 port. The capturing conditions were controlled by the physician reflecting the clinical conditions of an operation.

### Recording of testing targets

The testing targets were obtained from the Edmund Industrial Optics Company [25]. The general purpose of a test pattern is to determine the true color balance or optical density of any color system. It is an industry standard that provides a non-subjective comparison with a test pattern of 24 carefully prepared coloured squares. Each square in the pattern represents a natural color like the human skin, foliage, blue sky, etc. Testing images were captured at optimum illumination and focusing based on the experience of the physician, using the camera and the telescope under investigation. Following the above procedure we captured and saved the medical video (AVI format) of the testing palette and then extracted TIFF images of the 24 color squares. The corresponding targets were digitally generated based on the data given by the Edmund Optics Company [25] as the ground truth of the experiment. RGB values for some of the testing targets provided by the manufacturer are given in Table 1 and Appendix A.

**Table 1:** RGB digital values for some of the testing targets given by the Edmund Industrial Optics company [25].

**Color**	**R**	**G**	**B**
Yellow	255	217	0
Red	203	0	0
Light Green	140	253	153
Blue	0	0	142
White	255	255	255
Black	0	0	0
Blue Sky	97	119	171
Foliage	90	103	39

### Color correction algorithm

Most of the cameras have a nonlinear relationship between the signal voltage and the light intensity [26-28]. We assume that the recorded image intensity is a function of a simple linear model prior to separable non-linear gamma distortion. This model is compatible with the general model reported in Fig. 1 of [28]. We write:

$$\left[\begin{array}{c} R_{p}\\ G_{p}\\ B_{p} \end{array}\right]=\left[\begin{array}{ccc} a_{11} & a_{12} & a_{13}\\ a_{21} & a_{22} & a_{23}\\ a_{31} & a_{32} & a_{33} \end{array}\right]\left[\begin{array}{c} R_{in}\\ G_{in}\\ B_{in} \end{array}\right]+\left[\begin{array}{c} k_{1}\\ k_{2}\\ k_{3} \end{array}\right]$$

where: [*R*_*in *_*G*_*in *_*B*_*in*_]^*T *^denotes the red (*R*_*in*_), green (*G*_*in*_), and blue (*B*_*in*_) components of the target image intensity (values in Table 1, also see Appendix A), and [*R*_*p *_*G*_*p *_*B*_*p*_]^*T *^denotes the transformed components of the image intensity after capturing testing targets using the medical camera. The processed components are derived from the input image intensity components through multiplication by a linear **A **and a constant offset vector **k**. We then have a gamma model for the non-linear gamma relationship to the recorded image (components: *R*_*out*_, *G*_*out*_, *B*_*out*_):

$$\begin{array}{ccc} R_{out} & = & 255{\left(\frac{R_{p}}{255}\right)}^{\gamma_{R}}\\ G_{out} & = & 255{\left(\frac{G_{p}}{255}\right)}^{\gamma_{G}}\\ B_{out} & = & 255{\left(\frac{B_{p}}{255}\right)}^{\gamma_{B}}. \end{array}$$

To compute all the parameters of the model, we use non-linear least squares (see *lsqnonlin *function in MATLAB [29]) by solving equations (1) and (2) for known target images. We estimate matrices **A**, **k **and the gamma values, *γ*_*R*_, *γ*_*G*_, *γ*_*B *_for each color component. To recover the original, target image color components, we invert the color transformations given in equations (1) and (2). Appendix A presents details on the gamma correction procedure.

### Capturing video from experimental tissue in panoramic vs close up views

**Figure 2:**
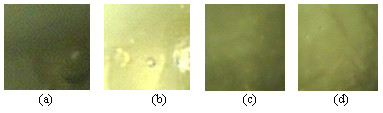
ROIs from the calf endometrium under different viewing conditions: (a) panoramic, (b) close up, (c) angle 1 and (d) angle 2.

A total of 40 images (20 panoramic, and 20 close up) were captured from experimental tissue from two calf endometria with the telescope at 3 and 5 cm for panoramic and close up views respectively (see Figures 2a and 2b).

A similar experiment was repeated using tissue of a chicken cavity. A total of 200 images (100 panoramic, and 100 close up) were captured from 10 chickens under the same viewing conditions as above.

### Capturing video from tissue at two different consecutive angle views

Similar to the previous experiment, a total of 40 images (20 at angle 1 and 20 at angle 2, with 3 degrees of difference) were captured from two calf endometria (see Figures 2c and 2d). The same experiments were carried out for the chicken cavity where a total of 200 images from 10 chicken cavities were captured at angle 1 and angle 2.

### Capturing video from the endometrium

The physician guides the telescope connected to a camera inside the uterus in order to investigate suspicious lesions of cancer. First, he/she investigates the anatomy of the organ and second, in panoramic view, he/she searches for suspicious areas. When a suspicious area is identified the physician switches to close up mode. This procedure is considered to be the standard procedure for identifying ROIs.

A total of 40 videos were recorded from 40 subjects from the endometrium. From these videos, 418 ROIs of 64 × 64 pixels were cropped and classified into two categories: (i) normal (N = 209) and (ii) abnormal (N = 209) ROIs based on the opinion of the physician and the histopathological examination.

### RGB to gray scale level transformation

All ROIs of 64 × 64 pixels were extracted from the tissue videos by the physician, for the purpose of clinical endoscopy imaging.

The RGB images were transformed to gray scale using

*Y *= (0.299 *R *+ 0.587 *G *+ 0.114 *B*)

where *Y *is the intensity image.

### Multiscale analysis

**Figure 3:**
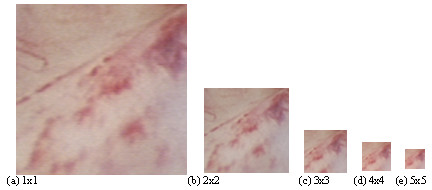
Multiscale analysis of an endometrium image of 128 × 128 pixels, (a) original image, and (b)–(e) downsampled images at rates 2 × 2 to 5 × 5 respectively.

In multiscale image analysis, an image is analyzed at different resolutions, revealing different characteristics at each resolution [30]. At low resolutions, only the larger image features are visible. In contrast, at high resolutions, finer texture features, as well as noise, are also visible. We are interested in identifying a particular range of scales where we have objects of diagnostic interest. To this end, we consider a variety of downsampling rates from 2 × 2 to 10 × 10. We note that at each rate, we have a complete representation of the input image at a number of bands that is proportional to the downsampling rate (4 for 2 × 2, 9 for 3 × 3, 100 for 10 × 10, etc). Yet, most of the image energy is almost always concentrated in the low-pass band, the one resulting from applying lowpass filtering in each direction (for separable designs), followed by downsampling. We thus focus our attention on the low-bands resulting from downsampling rates from 2 × 2 to 10 × 10, respectively. Figure 3, presents a real image from the endometrium in 1 × 1 up to 5 × 5 scales.

**Figure 4:**
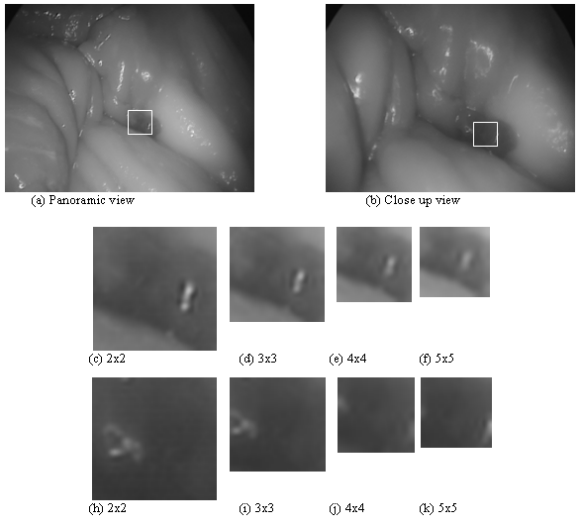
Original gray scale image from chicken cavity with ROIs shown in square area with white perimeter, (a) panoramic view and (b) close up view. Chicken cavity ROIs with downsampled images, (c) – (f) panoramic views at scales 2 × 2 to 5 × 5, respectively and (f) – (k) close up views at 2 × 2 to 5 × 5, respectively.

Figure 4 illustrates the original chicken cavity images for panoramic and close up views (ROI images after multiscale analysis for the scales 2 × 2 to 5 × 5). There are significant differences among the resized images, depending on the downsampling rates. However, as expected, the larger texture features appear in all images, while, as the downsampling rate increases, the finer texture features begin to disappear.

### Feature extraction

Texture features were extracted from the segmented ROI images in order to characterize tissue captured under different viewing conditions, as well as to differentiate between normal and abnormal tissue. A total number of 26 texture features were extracted from endoscopic images (described next). These feature sets were also successfully used in numerous previous works in texture analysis [31]. Some of the features used capture complementary textural properties, however, features that were highly dependent or similar with features in other feature sets, were identified through statistical analysis and eliminated. The ROI color images were transformed into grayscale images and the following texture features were computed:

#### Statistical Features (SF)

SF features describe the gray level histogram distribution without considering spatial dependence [15]. The following SF texture features were computed: 1) Mean, 2) Variance, 3) Median, 4) Energy, 5) Skewness, 6) Kurtosis, and 7) Entropy.

#### Spatial Gray Level Dependence Matrices (SGLDM)

The spatial gray level dependence matrices as proposed by Haralick *et al.*[16] are based on the estimation of the second-order joint conditional probability density functions that two pixels (*k*, *l*) and (*m*, *n*) with distance *d *in direction specified by the angle θ, have intensities of gray level (*i*) and gray level (*j*). Based on the estimated probability density functions, the following texture measures out of the 13 proposed by Haralick *et al.*[16] were computed: 1) Angular second moment (ASM), 2) Contrast, 3) Correlation, 4) Auto-Correlation, 5) Variance, 6) Inverse Difference Moment, 7) Entropy, 8) Sum Entropy, 9) Sum Average, 10) Sum Variance, 11) Difference Entropy etc. For a selected distance *d *(in this work *d *= 1 was used), and for angles *θ *= 0°, 45°, 90° and 135° we computed four values for each of the above texture measures. The above features were calculated for displacements δ = (0,1), (1,1), (1,0), (1,-1), where δ = (Δx, Δy), and their range of values were computed.

#### Gray level difference statistics (GLDS)

The GLDS algorithm [17] is based on the assumption that useful texture information can be extracted using first order statistics of an image. The algorithm is based on the estimation of the probability density *p*_*δ *_of image pixel pairs at a given distance δ = (Δx, Δy), having a certain absolute gray level difference value. For any given displacement δ = (Δx, Δy), let *f*_*δ*_(*x*, *y*) = |*f*(*x*, *y*) - *f*(*x *+ Δ_*x*_, *y *+ Δ_*y*_)|. Let *p*_*δ *_be the probability density of *f*_*δ*_(*x*, *y*). If there are *m *gray levels, this has the form of an *m*-dimensional vector whose *i*th component is the probability that *f*_*δ*_(*x*, *y*) will have value (*i*). If the picture *f *is discrete, it is easy to compute *p*_*δ *_by counting the number of times each value of *f*_*δ*_(*x*, *y*) occurs, where Δx and Δy are integers. Coarse texture images result in low gray level difference values, whereas, fine texture images result in inter-pixel gray level differences with great variances. Features were estimated for the following distances: δ = (d,0), (d,d), (-d,d), (0,d). A good way to analyze texture coarseness is to compute, for various magnitudes of δ, some measure of the spread of values in *p*_*δ *_away from the origin. Some of the features that were computed are: 1) Mean, 2) Entropy, 3) Contrast, and 4) Energy.

### Statistical analysis

The Wilcoxon rank sum test was applied [32] to investigate if the texture features have significant statistical difference for different viewing conditions (panoramic vs close up, angle 1 vs angle 2) and between texture features extracted before and after gamma correction at a = 0,05. The Wilcoxon test returns a p-value, which represents the probability of observing the given data by chance if the medians are equal. Small values of p imply that the null hypothesis should be rejected [33].

## Results

### Color correction algorithm

The Circon IP4.1 endoscopy camera was used for capturing video from both the testing targets and tissues. In these experiments, the color correction algorithm was run using the recorded test targets and the ground truth images as supplied by Edmund Optics Company. The computed color correction parameters were then used for correcting the images.

Table 2 tabulates the **A, k **and **γ **values of the R, G, B channels for three different experiments as well as their median values. It is clearly shown that a variability exists between the **A, k**, and **γ **values for these experiments. The variability documented in Table 2 motivated us to investigate it further. A database of 209 normal and 209 abnormal ROIs of the endometrium recorded from 40 women was analysed. Images were corrected, using different combinations of the **A**, **k**, and **γ **values and their corresponding texture features were computed. Neural network models were trained to classify 100 normal and 100 abnormal endometrium images. The rest of the cases were used for evaluating the performance of the models. The percentage of correct classifications score was computed for the evaluation set. It was found that the texture features computed with the median values of **A**, **k **and **γ **for the three experiments gave the highest score. The results of these experiments are reported in detail in another study [21]. It was thus decided to use, the median values of **A**, **k **and **γ **in this study as well. The median gamma values for the three channels (*γ*_*R *_= 1,078 *γ*_*G *_= 1,046, *γ*_*B *_= 1,040) were very close to unit values.

**Table 2:** Gamma correction parameters A, k and γ for three different experiments and their median values

**A **matrix	No Correction	Exp 1	Exp 2	Exp 3	Median values for Exps 1, 2, 3
a_11_	1	0,827	0,927	0,975	0,927
a_12_	0	0,065	0,011	0,105	0,065
a_13_	0	0,042	0,004	0,104	0,042
a_21_	0	0,065	0,011	0,105	0,065
a_22_	1	0,780	0,935	0,895	0,895
a_23_	0	0,071	0,062	0,134	0,071
a_31_	0	0,042	0,004	0,104	0,042
a_32_	0	0,044	0,032	0,023	0,032
a_33_	1	0,868	1,011	1,044	1,011

**k **matrix					

k_11_	0	7,693	1,101	-1,673	1,101
k_21_	0	10,083	2,090	0,528	2,090
k_31_	0	-8,161	1,598	-5,689	-5,689

**γ **matrix					

γ_R_	1	1,285	1,078	1,038	1,078
γ_G_	1	1,220	1,046	0,999	1,046
γ_B_	1	1,180	0,971	1,040	1,040

Table 3 tabulates the MSE using the Circon IP4.1 endoscopy camera for the uncorrected images (first column) and the gamma-corrected images (second column). It is clear that the MSE drops significantly after gamma correction.

**Table 3:** MSE for three experiments for uncorrected (first column) and gamma corrected (second column) using the median values of the endoscopy output image before and after calibration (for calibrated targets)

MSE for each Channel		
Channels	$MSE_{org-cam}=\frac{1}{NM}\sum_{i,j=1}^{N,M}{\left(I_{org_{i,j}}-I_{camera_{i,j}}\right)}^{2}$	$MSE_{org-cor}=\frac{1}{NM}\sum_{i,j=1}^{N,M}{\left(I_{org_{i,j}}-I_{cor_{i,j}}\right)}^{2}$

Exp 1		

Red	3342	482
Green	2088	350
Blue	1228	415

Exp 2		

Red	1605	570
Green	2180	443
Blue	2545	670

Exp 3		

Red	3301	578
Green	1973	415
Blue	3035	316

Mean values of Exp 1, 2 and 3		

Red	2749	543
Green	2080	403
Blue	2269	467

### Capturing video from experimental tissue in close up vs panoramic views

The results of the statistical analysis in the close up vs the panoramic view (using experimental tissues) indicates the variability due to the use of different viewing conditions. For this experiment, we use calf endometria, in an environment that is similar to actual operating conditions.

**Figure 5:**
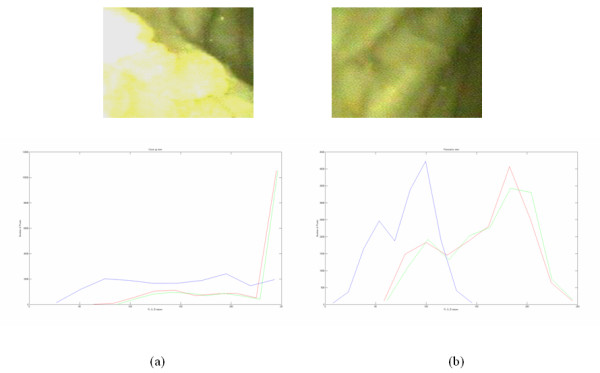
Histogram plots for R, G and B channels for calf endometrium for (a) close-up and (b) panoramic views (after gamma correction).

Figure 5 illustrates ROIs and their corresponding R, G, B histograms from the calf endometrium in panoramic vs close up views after gamma correction. The pixel distribution is similar, with slightly higher values for the panoramic view.

Table 4 tabulates the texture features and the statistical analysis for panoramic vs close up views for the original images. The columns present the P5^th^, P25^th^, P50^th ^(median), P75^th ^and the P95^th ^percentiles for each texture feature. Also the Wilcoxon rank sum test was used at a = 0,05 to investigate if there was significant difference (H = 1) or not (H = 0) between the different views.

**Table 4:** Percentile values of the texture features for the panoramic vs close up views of the calf endometrium before gamma correction (for N = 20).

	**Panoramic View**					**Close up View**					
	**P5%**	**P25%**	**P50%**	**P75%**	**P95%**	**P5%**	**P25%**	**P50%**	**P75%**	**P95%**	**H**

**SF**											

**Mean**	36,49	42,42	50,73	58,72	89,41	39,50	41,91	80,65	125,33	149,09	0
**Variance**	55,78	108,13	201,17	244,69	431,18	55,61	259,80	633,07	1708,22	1795,52	0
**Median**	36,86	42,62	50,29	62,08	88,36	40,16	43,51	83,46	131,32	161,01	0
**Mode**	40,00	42,00	51,00	69,00	88,00	40,00	53,00	101,00	155,00	169,00	0
**Skewness**	-0,42	-0,21	-0,12	0,23	0,28	-0,91	-0,40	-0,27	-0,03	0,26	0
**Kurtosis**	2,09	2,31	2,56	2,66	2,74	1,89	1,91	2,33	2,77	3,33	0
**Entropy**	3,39	3,74	4,03	4,06	4,39	3,40	4,08	4,51	4,94	5,00	0

**SGLDM**											

**Contrast**	27,49	27,50	28,04	30,00	32,17	27,71	28,06	29,68	34,68	62,59	0
**Correlation**	0,75	0,87	0,92	0,94	0,97	0,73	0,95	0,97	0,98	0,99	0
**Variance**	55,54	106,97	199,49	243,39	425,94	55,08	258,21	626,46	1688,81	1787,09	0
**Homogeneity**	0,20	0,20	0,21	0,21	0,21	0,19	0,19	0,21	0,21	0,21	0
**Entropy**	6,33	6,72	7,04	7,13	7,38	6,38	7,08	7,55	7,98	8,10	0

**GLDS**											

**Homogeneity**	0,20	0,20	0,21	0,21	0,21	0,19	0,19	0,21	0,21	0,21	0
**Contrast**	27,50	27,50	28,04	30,01	32,17	27,71	28,06	29,68	34,68	62,54	0
**Energy**	0,10	0,10	0,10	0,10	0,10	0,08	0,09	0,10	0,10	0,10	0
**Entropy**	2,44	2,44	2,45	2,48	2,51	2,44	2,45	2,47	2,55	2,74	0
**Mean**	4,13	4,13	4,17	4,33	4,46	4,14	4,18	4,25	4,62	5,50	0

**Figure 6:**
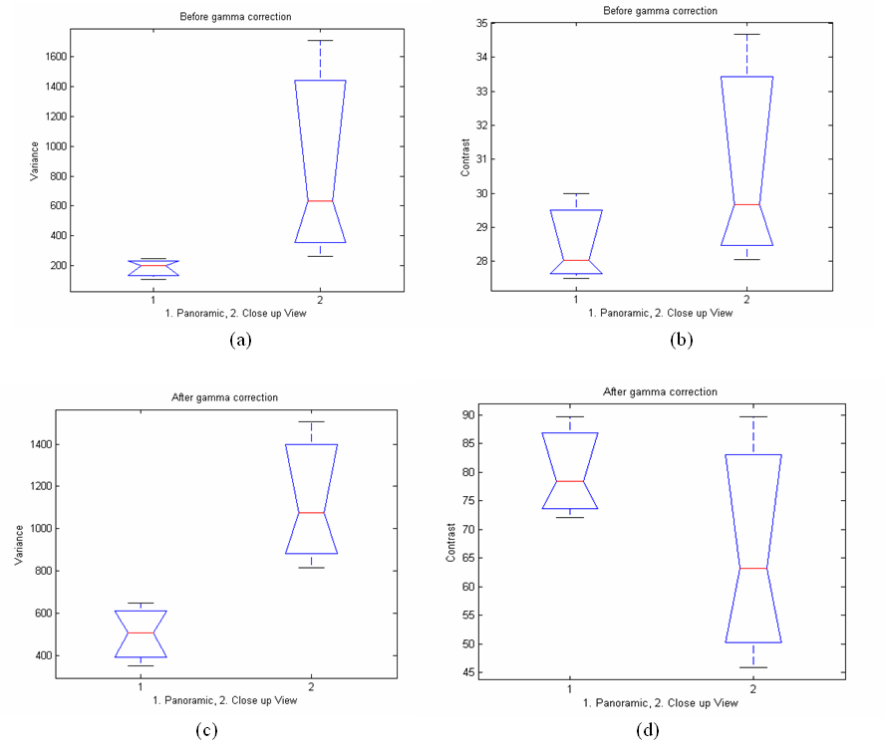
Box plots of selected texture features of experimental tissue (calf endometrium) for panoramic and close up views before and after gamma correction. Plots (a) and (b) present SF variance and SGLDM contrast features before gamma correction respectively. Plots (c) and (d) present the same texture features after applying gamma correction. (The notched box shows the median, lower and upper quartiles and confidence intervals around the median for each feature. The dotted lines connect the nearest observations within 1.5 of the inter-quartile range (IQR) of the lower and upper quartiles.)

Prior to gamma correction, we have found that there was no significance difference between features computed from the panoramic and close-up views. We note that from the table, we can see that we do appear to have some significant differences in particular features. For example, the P50% for the SF mean feature in the close up view is approximately 81 compared to an approximate value of 51 for the panoramic view. Also the SF variance in the close up view is higher (633, see Figure 6), compared to the value for the panoramic view (201). Nevertheless, both features exhibit large variability around these median values and it is for this reason that the Wilcoxon test found no significant differences. On the other hand, the SGLDM contrast is 30 in the close up and 28 in the panoramic views respectively, maintaining fairly constant median values. Similarly, SGLDM homogeneity is 0,21 for close up and 0,21 for the panoramic view. The SGLDM entropy feature is approximately the same for both views, while (like for the SF variance) the variance in the close up view is higher than in the panoramic view (199 vs 626 respectively).

Table 5 tabulates the texture features and the statistical analysis for panoramic vs close up views after applying the gamma correction algorithm. The results show that there is the same trend in the texture features as in Table 4. The SF variance, in the close up view is higher than in the panoramic view, the SF median feature is also following the same trend and the SF entropy is the same in both views. The SGLDM contrast is 63 in close up and 78 in panoramic views respectively. Also the SGLDM homogeneity varies very little in both views. After inspecting the other SGLDM and GLDS texture features, we can see that the SGLDM variance for the close up view is higher than for the panoramic view. On the other hand, SGLDM entropy varies very little between views.

**Table 5:** Percentile values of the texture features for the panoramic vs close up view of the calf endometrium after gamma correction (N = 20). Also statistical analysis is tabulated for comparing before vs after gamma correction for panoramic and close up views

											**Statistical Analysis**		
											
	**Panoramic View**					**Close up View**					**Panoramic vs Close up after gamma**	**Before vs After Gamma for Panoramic views**	**Before vs After Gamma for close up views**
	**P5%**	**P25%**	**P50%**	**P75%**	**P95%**	**P5%**	**P25%**	**P50%**	**P75%**	**P95%**	**H**	**H**	**H**

**SF**													

**Mean**	86,48	97,19	110,26	122,79	169,87	92,16	95,16	151,04	212,50	223,83	0	1	0
**Variance**	166,31	351,08	506,72	646,23	1094,79	172,50	815,22	1075,93	1505,68	2731,20	0	0	0
**Median**	87,80	97,59	110,49	129,64	168,85	93,66	99,32	159,48	227,37	242,01	0	1	0
**Mode**	92,00	97,00	114,00	139,00	170,00	97,00	119,00	182,00	244,00	245,00	0	1	0
**Skewness**	-0,51	-0,37	-0,28	0,05	0,13	-1,98	-0,84	-0,44	-0,38	0,03	0	0	0
**Kurtosis**	2,18	2,39	2,64	2,68	2,88	1,74	2,06	2,58	2,76	5,88	0	0	0
**Entropy**	3,91	4,30	4,45	4,55	4,84	3,89	3,95	4,53	4,82	4,86	0	1	0

**SGLDM**													

**Contrast**	65,89	72,00	78,41	89,66	91,19	41,61	45,87	63,18	89,62	90,42	0	1	1
**Correlation**	0,75	0,87	0,92	0,94	0,97	0,74	0,94	0,97	0,98	0,99	0	0	0
**Variance**	165,55	346,97	502,15	642,44	1081,54	170,73	809,78	1061,78	1487,91	2721,95	0	1	0
**Homogeneity**	0,13	0,13	0,14	0,14	0,15	0,13	0,13	0,20	0,27	0,44	0	1	0
**Entropy**	7,33	7,78	7,83	8,03	8,21	6,09	7,29	7,57	8,07	8,20	0	1	0

**GLDS**													

**Homogeneity**	0,13	0,13	0,14	0,14	0,15	0,13	0,13	0,20	0,27	0,44	0	1	0
**Contrast**	65,90	72,01	78,42	89,67	91,21	41,61	45,83	63,17	89,64	90,43	0	1	1
**Energy**	0,06	0,06	0,06	0,06	0,07	0,06	0,06	0,08	0,10	0,19	0	1	0
**Entropy**	2,86	2,90	2,94	3,01	3,01	2,21	2,60	2,80	3,00	3,01	0	1	0
**Mean**	6,40	6,67	6,95	7,46	7,52	3,37	4,54	5,90	7,43	7,45	0	1	0

Table 5 also tabulates the results of the Wilcoxon rank sum test between the panoramic and close up views before and after gamma correction. Figure 6 illustrates box plots of the SF variance and SGLDM contrast in panoramic vs close up views before and after applying gamma correction.

We have found significant differences when comparing texture feature values before and after gamma correction. These differences are fairly dramatic for texture feature values from the panoramic views, and somewhat less pronounced for the close up views. Furthermore, as before, after gamma correction, there were no significant differences between the texture features from the close up and panoramic views. These observations suggest that gamma correction is an essential and required step for reducing texture feature variability due to varying viewing conditions.

We also repeated these experiments using the chicken cavities, under the same viewing conditions and the same medical equipment. The results were very similar as for the calf endometria and we will not repeat them here.

### Capturing video from experimental tissue in two different consecutive angle views

We now present statistical analysis results for texture feature values extracted from different angles. Here, we note that gamma correction did not seem to affect the results.

**Figure 7:**
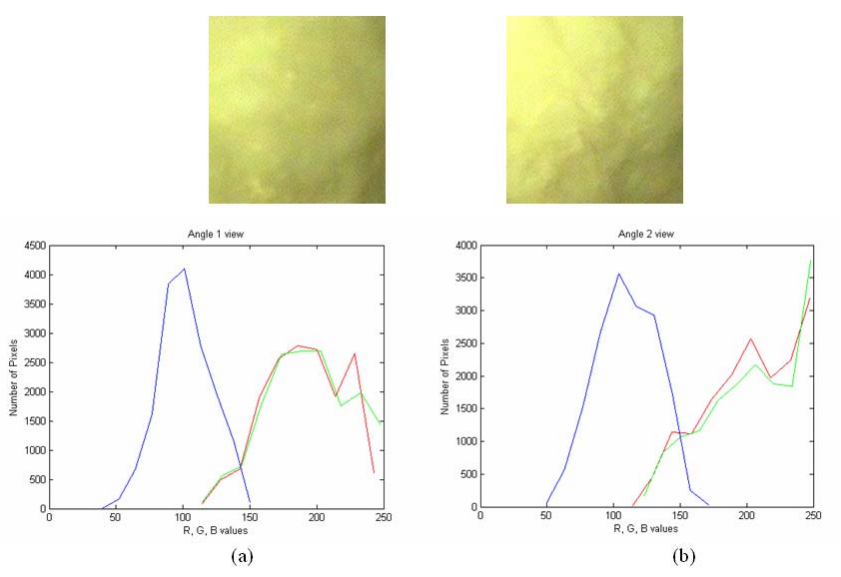
Histogram plots for R, G and B channels for calf endometrium for (a) angle 1 and (b) angle 2 views (after gamma correction).

Figure 7 presents the ROIs from calf endometria captured in (a) and (b) after gamma correction (for two different viewing angles).

Tables 6 and 7 tabulate the texture features and the statistical analysis form ROIs that were captured from two different consecutive angles of 3 degrees difference for the uncorrected and gamma corrected images respectively. In Table 6 the P50%, for SF variance is 181 for angle 1 view, is reduced to 93 in angle 2 view, while the SF median is the same 43 vs 45 respectively. Also the SF entropy is the same in both angle views. The SGLDM variance is much higher in angle 1 view, compared with that of angle 2 view. Note, that the entropy for the SF, SGLDM and GLDS feature sets is the same for both angle 1 and angle 2 views.

**Table 6:** Percentile values of the texture feature values from two consecutive angles (differing by 3 degrees) for the calf endometrium before gamma correction (for N = 20).

	**Angle 1 View**					**Angle 2 View**					
	**P5%**	**P25%**	**P50%**	**P75%**	**P95%**	**P5%**	**P25%**	**P50%**	**P75%**	**P95%**	**H**

**SF**											

**Mean**	31,98	37,07	43,43	92,53	182,63	27,79	34,06	42,64	49,79	64,03	0
**Variance**	49,56	93,37	181,76	343,80	476,76	58,52	63,58	93,85	263,72	448,90	0
**Median**	32,10	37,23	43,74	89,29	182,07	26,32	33,89	45,63	52,12	70,77	0
**Mode**	40,00	40,00	42,00	87,00	183,00	22,00	34,75	50,00	61,25	80,00	0
**Skewness**	-0,35	-0,14	-0,06	0,07	0,40	-0,47	-0,38	-0,24	0,08	0,39	0
**Kurtosis**	1,94	2,34	2,57	2,81	3,44	1,63	2,13	2,30	2,50	2,67	0
**Entropy**	3,36	3,63	3,94	4,28	4,41	3,44	3,46	3,62	4,03	4,18	0

**SGLDM**											

**Contrast**	27,55	28,33	28,77	32,80	37,37	26,86	28,17	28,62	29,18	30,29	0
**Correlation**	0,71	0,83	0,92	0,94	0,97	0,74	0,78	0,85	0,94	0,97	0
**Variance**	49,31	92,78	180,49	340,48	471,72	58,22	63,18	93,61	261,78	448,53	0
**Homogeneity**	0,20	0,21	0,21	0,21	0,21	0,21	0,21	0,21	0,21	0,21	0
**Entropy**	6,33	6,61	6,95	7,31	7,42	6,41	6,45	6,60	7,03	7,15	0

**GLDS**											

**Homogeneity**	0,20	0,21	0,21	0,21	0,21	0,21	0,21	0,21	0,21	0,21	0
**Contrast**	27,56	28,34	28,78	32,80	37,35	26,86	28,17	28,62	29,19	30,29	0
**Energy**	0,10	0,10	0,10	0,10	0,10	0,10	0,10	0,10	0,10	0,10	0
**Entropy**	2,44	2,45	2,46	2,49	2,52	2,43	2,45	2,46	2,46	2,48	0
**Mean**	4,15	4,20	4,24	4,37	4,42	4,09	4,18	4,22	4,25	4,33	0

**Table 7:** Percentile values of the texture features from two consecutive angles for the calf endometrium after gamma correction (N = 20). Also statistical analysis results is tabulated for comparing before vs after gamma correction for angle 1 and angle 2 views.

											**Statistical Analysis**		
											
	**Angle 1 View**					**Angle 2 View**					**Angle 1 vs Angle 2 after gamma**	**Before vs After Gamma for Angle 1**	**Before vs After Gamma for Angle 2**
	**P5%**	**P25%**	**P50%**	**P75%**	**P95%**	**P5%**	**P25%**	**P50%**	**P75%**	**P95%**	**H**	**H**	**H**

**SF**													

**Mean**	33,68	38,98	45,56	94,08	181,43	29,47	35,91	44,78	51,99	66,35	0	0	0
**Variance**	51,96	97,59	199,06	314,46	484,39	62,07	66,4	104	276,13	456,75	0	0	0
**Median**	33,98	39,25	45,91	90,93	180,9	27,96	35,77	47,91	54,35	73,26	0	0	0
**Mode**	42	42,75	44	88,25	182	24	35,25	51	63,75	84	0	0	0
**Skewness**	-0,38	-0,18	-0,1	0,05	0,36	-0,51	-0,41	-0,26	0,05	0,36	0	0	0
**Kurtosis**	1,95	2,34	2,59	2,85	3,49	1,64	2,1	2,34	2,51	2,66	0	0	0
**Entropy**	3,38	3,65	3,98	4,23	4,42	3,47	3,48	3,67	4,05	4,19	0	0	0

**SGLDM**													

**Contrast**	27,81	28,98	30,4	31,05	32,57	26,92	27,91	29,12	30,7	31,34	0	0	0
**Correlation**	0,72	0,84	0,92	0,94	0,97	0,75	0,79	0,85	0,94	0,97	0	0	0
**Variance**	51,69	96,95	197,6	311,39	479,23	61,75	65,99	103,74	273,98	456,32	0	0	0
**Homogeneity**	0,2	0,2	0,21	0,22	0,23	0,2	0,2	0,21	0,21	0,21	0	0	0
**Entropy**	6,37	6,64	7,02	7,21	7,42	6,43	6,49	6,68	7,05	7,15	0	0	0

**GLDS**													

**Homogeneity**	0,21	0,21	0,22	0,22	0,23	0,21	0,21	0,22	0,22	0,22	0	0	0
**Contrast**	27,81	28,98	30,4	31,04	32,55	26,92	27,91	29,12	30,7	31,34	0	0	0
**Energy**	0,1	0,1	0,1	0,1	0,11	0,1	0,1	0,1	0,1	0,1	0	0	0
**Entropy**	2,44	2,45	2,47	2,48	2,48	2,43	2,44	2,46	2,49	2,5	0	0	0
**Mean**	4,12	4,16	4,27	4,31	4,35	4,09	4,16	4,26	4,37	4,41	0	0	0

After applying gamma correction we extract the texture features as shown in Table 7. The SF variance is higher for angle 1 view and is reduced in the angle 2 view. Also the SF median feature has the same values for both angle views and the SF entropy remains the same for both views. The SGLDM variance is following the same trend as above and the entropy for SGLDM and GLDS is approximately the same.

**Figure 8:**
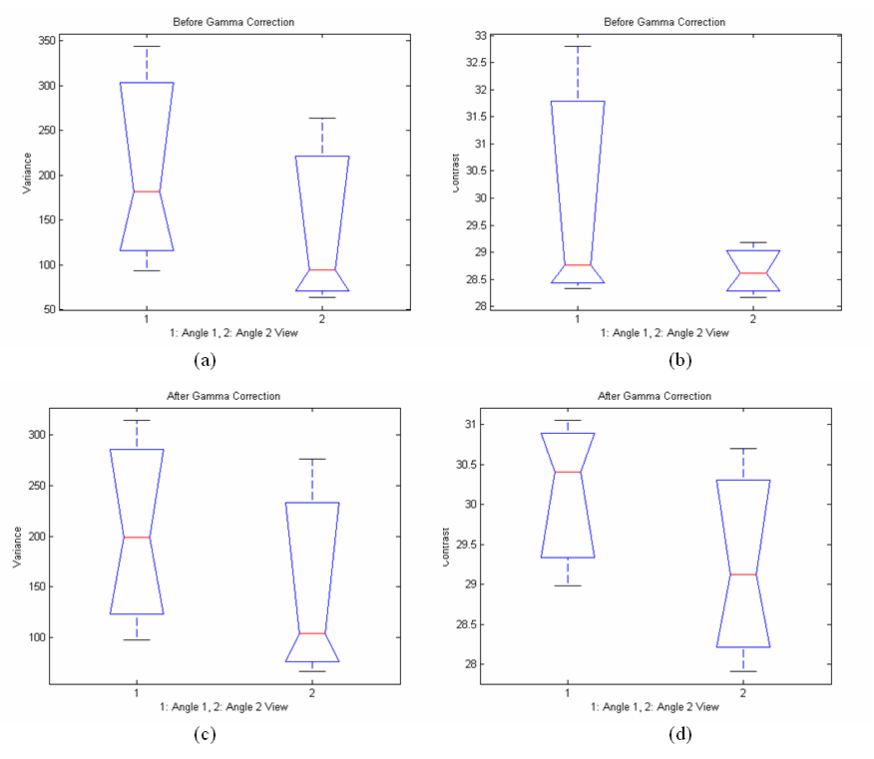
Box plots of selected texture features of experimental tissue (calf endometrium) for Angle 1 and Angle 2 views before and after gamma correction. Plots (a) and (b) present SF variance and SGLDM contrast features before gamma correction respectively. Plots (c) and (d) present the same texture features after applying gamma correction. (The notched box shows the median, lower and upper quartiles and confidence interval around the median for each feature. The dotted lines connect the nearest observations within 1.5 of the inter-quartile range (IQR) of the lower and upper quartiles.)

Table 7 tabulates the results of the texture features analysis and the Wilcoxon rank sum test when comparing characteristics between the uncorrected images from angle 1 and angle 2 vs corrected images from the same angle views. Figure 8 also shows results for SF variance and SGLDM contrast. Texture features such as entropy, variance and the mean are approximately the same before and after gamma correction for both cases. Images after gamma correction are very close to the original uncorrected images, as judged by the physician. As shown, there is no significant difference between the texture features values. It is clear that there are no significant differences between texture feature values from different angles, whether we apply gamma correction or not.

As before, we also repeated these experiments using the chicken cavities, under the same viewing conditions and the same medical equipment. The results were again very similar as for the calf endometria and we will not repeat them here.

### Multiscale analysis

For completeness, we repeat the analysis at multiple scales. The analysis was applied to ROIs cropped from 128 × 128 to 22 × 22 after resample the original images. This was done from 1 × 1 until the 10 × 10 scales. Notice that the results can be monitored until the 3 × 3 scale because in higher analysis the images are destroyed visually and the information that is included can not be used. The results were also performed on the chicken cavity datasets.

**Figure 9:**
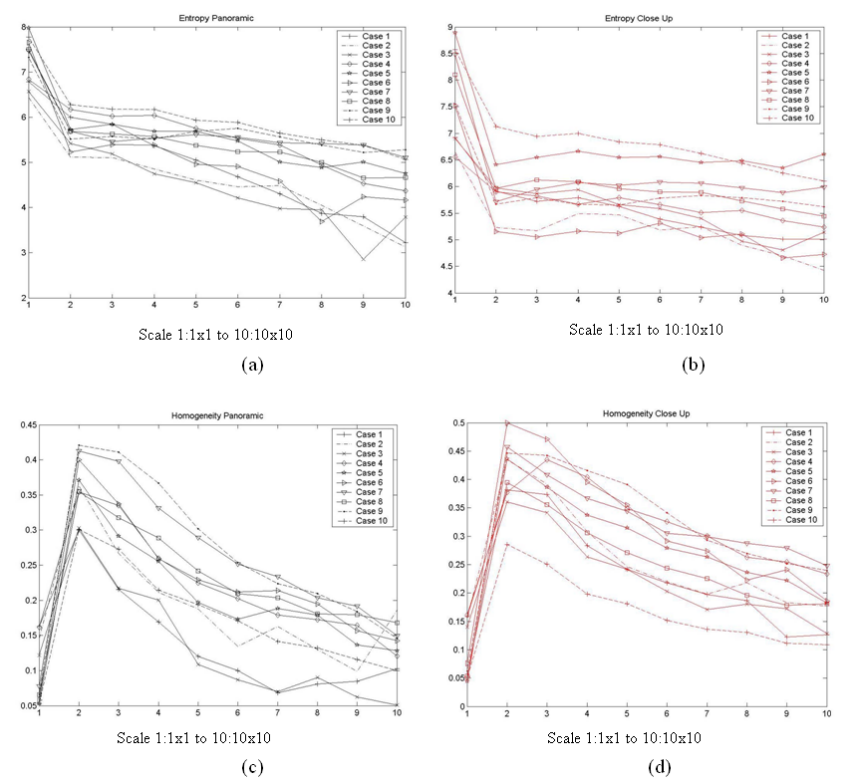
Texture feature value variability for the panoramic and close-up views as a function of scale, for SGLDM entropy and GLDS homogeneity.

**Figure 10:**
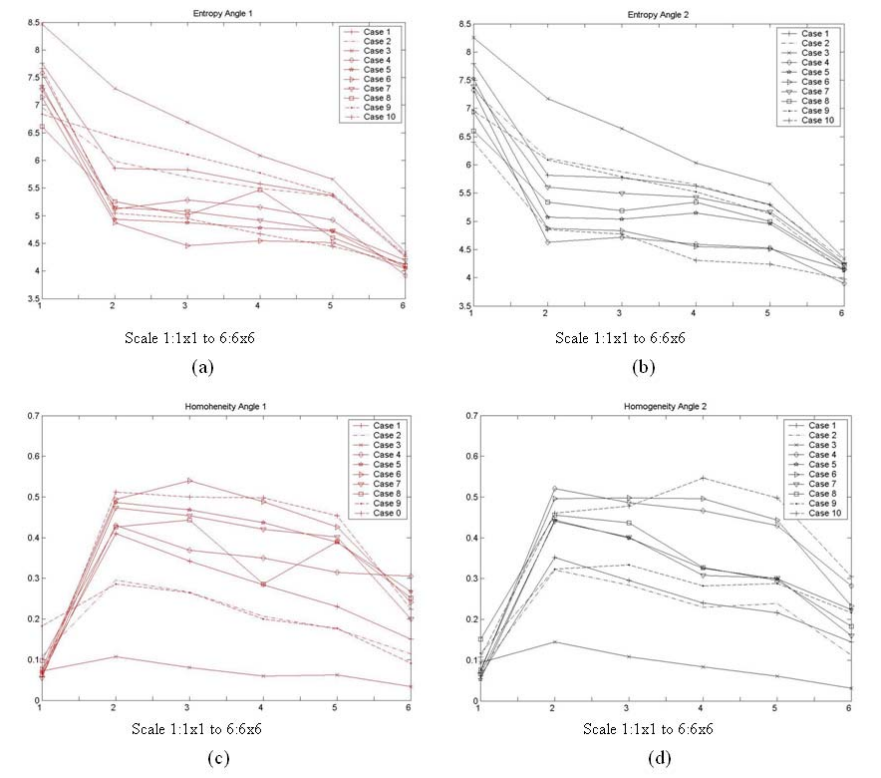
Texture feature value variability for the angle 1 and angle 2 views as a function of scale for SGLDM entropy and GLDS homogeneity.

Figures 9 and 10 present graphically how some texture feature values (SGLDM entropy and GLDS homogeneity) vary as a function of scales 1 × 1 to 10 × 10, 1 × 1 to 6 × 6 respectively and viewing conditions. Here, recall that the analysis was carried out at the lower scales only.

In the entropy plots (Figs. 9(a)–(b) and 10(a)–(b)), we can see an overall downward trend. On the contrary, for the homogeneity plots (Figs. 9(c)–(d) and 10(c)–(d)), we can see a dramatic rise that is followed by a slow downward trend at higher scales. After visual inspection, we have concluded that the lower homogeneity values at the 1 × 1 is due to high-frequency instrument noise that is removed by the lowpass filtering associated with the computation of the lower-scales at 2 × 2. At higher scales, we have a smaller number of pixels that represent the same ROI as for the original 1 × 1 scale. The dramatic reduction in the number of pixels (100:1 at 10 × 10 and 6 × 6 respectively) results in a reduction in entropy. On the other hand, the fact that a significantly smaller number of pixels are used to represent the same ROI results in a reduction of homogeneity.

### Analysis of images of human endometria

In this subsection we present results from the statistical analysis of ROIs extracted from human endometria. The results are summarized in Tables 8 and 9.

**Table 8:** Percentile values of the texture features and statistical analysis of normal (N = 209) vs abnormal (N = 209) ROIs of human endometrium extracted from 40 subjects. Statistical analysis was carried out before gamma correction at a ≤ 0,05.

	**Normal ROIs**					**Abnormal ROIs**					
	**P5%**	**P25%**	**P50%**	**P75%**	**P95%**	**P5%**	**P25%**	**P50%**	**P75%**	**P95%**	**H**

**SF**											

**Mean**	87,37	116,29	136,80	156,64	192,98	75,32	108,04	124,99	152,15	195,89	1
**Variance**	15,01	36,43	67,25	155,76	351,77	33,38	82,60	142,85	287,53	617,21	1
**Median**	87,66	116,81	136,96	157,58	192,24	75,41	106,67	123,21	153,35	197,99	1
**Mode**	85,95	116,00	136,00	158,00	188,05	74,00	103,50	125,00	159,00	202,10	1
**Skewness**	-1,00	-0,43	-0,11	0,14	0,58	-1,06	-0,43	-0,11	0,22	0,63	0
**Kurtosis**	1,93	2,26	2,63	3,08	4,28	1,81	2,22	2,61	3,07	4,68	0
**Energy**	0,02	0,03	0,04	0,05	0,08	0,01	0,02	0,03	0,03	0,05	1
**Entropy**	2,73	3,15	3,44	3,82	4,19	3,12	3,54	3,81	4,09	4,41	1

**SGLDM**											

**Contrast**	2,96	3,62	4,58	5,93	15,27	3,04	5,20	8,16	13,70	25,42	1
**Correlation**	0,85	0,93	0,96	0,98	0,99	0,91	0,95	0,97	0,98	0,99	1
**Variance**	14,75	35,61	65,93	154,00	344,69	32,83	81,91	140,56	280,55	605,91	1
**Homogeneity**	0,34	0,42	0,45	0,48	0,50	0,29	0,36	0,39	0,44	0,50	1
**Entropy**	4,65	5,11	5,48	6,01	6,49	5,10	5,64	6,04	6,44	6,79	1

**GLDS**											

**Homogeneity**	0,34	0,42	0,45	0,48	0,50	0,29	0,36	0,39	0,44	0,50	1
**Contrast**	2,95	3,62	4,57	5,92	15,23	3,04	5,19	8,15	13,67	25,36	1
**Energy**	0,16	0,22	0,24	0,26	0,28	0,13	0,17	0,20	0,23	0,28	1
**Entropy**	1,43	1,52	1,61	1,73	2,13	1,45	1,66	1,84	2,04	2,31	1
**Mean**	1,29	1,43	1,58	1,78	2,81	1,31	1,68	2,03	2,54	3,41	1

**Table 9:** Percentile values of the texture features and statistical analysis for normal (N = 209) vs abnormal (N = 209) ROIs of the endometrium extracted from 40 subjects. Statistical analysis was carried out after gamma correction but also between the normal/abnormal ROIs before and after gamma correction at a ≤ 0,05.

	**Normal ROIs**					**Abnormal ROIs**					**Normal vs Abnormal ROIs**	**Original vs Corrected Images For Normal ROIs**	**Original vs Corrected Images For Abnormal ROIs**
	**P5%**	**P25%**	**P50%**	**P75%**	**P95%**	**P5%**	**P25%**	**P50%**	**P75%**	**P95%**	**H**	**H**	**H**

**SF**													

**Mean**	110,11	138,44	156,06	173,91	204,36	98,48	129,37	144,65	170,48	206,06	1	1	1
**Variance**	13,23	29,44	54,63	127,94	286,63	31,33	66,9	124,39	223,33	492,3	1	1	1
**Median**	110,18	138,83	156,44	174,42	203,53	98,2	127,92	143,75	171,43	207,7	1	1	1
**Mode**	109,95	135,75	156	175	201,05	98	124	146,5	176	211,4	1	1	1
**Skewness**	-1,01	-0,46	-0,14	0,12	0,56	-1,14	-0,47	-0,14	0,18	0,62	0	0	0
**Kurtosis**	1,94	2,26	2,64	3,09	4,39	1,82	2,24	2,62	3,16	4,85	0	0	0
**Energy**	0,02	0,03	0,04	0,06	0,09	0,02	0,02	0,03	0,04	0,06	1	1	1
**Entropy**	2,66	3,02	3,34	3,68	4,09	3,11	3,44	3,74	3,99	4,32	1	1	1

**SGLDM**													

**Contrast**	2,54	3,1	3,82	4,87	12,27	2,55	4,82	7,04	10,99	21,94	1	1	1
**Correlation**	0,85	0,93	0,96	0,98	0,99	0,91	0,95	0,97	0,98	0,99	1	1	0
**Variance**	13,02	28,83	53,97	126,41	284,3	30,89	65,53	120,85	221,38	488,55	1	1	1
**Homogeneity**	0,37	0,45	0,48	0,5	0,53	0,31	0,38	0,42	0,46	0,53	1	1	1
**Entropy**	4,47	4,93	5,31	5,78	6,28	5,01	5,49	5,93	6,28	6,65	1	1	1

**GLDS**													

**Homogeneity**	0,37	0,45	0,48	0,5	0,53	0,31	0,38	0,42	0,46	0,53	1	1	1
**Contrast**	2,54	3,09	3,81	4,86	12,24	2,55	4,81	7,03	10,97	21,89	1	1	1
**Energy**	0,17	0,24	0,25	0,27	0,3	0,14	0,18	0,21	0,24	0,3	1	1	1
**Entropy**	1,37	1,45	1,54	1,64	2	1,37	1,63	1,77	1,96	2,24	1	1	1
**Mean**	1,18	1,33	1,44	1,63	2,45	1,19	1,62	1,89	2,31	3,16	1	1	1

Table 8 presents the texture features results before gamma correction. The non-parametric Wilcoxon rank sum test was used to decide if there is a significant difference between normal and abnormal ROIs at a = 0,05. The results indicate that there is a significant difference. Furthermore, as we can see in Table 8, the entropy values are preserved. From the table, it is clear that the median SGLDM contrast for abnormal cases is dramatically larger than the corresponding median value for the normal ROIs.

Table 9 presents comparative results after gamma correction. The results indicate that there are significant differences in the same texture features that were found in Table 8, before gamma correction. This shows that gamma correction did not reduce the discriminative power of the texture features. On the other hand, we can also see significant differences between the gamma-corrected and uncorrected images, indicating the importance of gamma correction.

## Discussion

For the standardized protocol, we propose a distance of 3 cm for close up examinations and a distance of 5 cm for panoramic examinations. We note that the viewing angle (the turning angle of the telescope) is much higher in case of laparoscopy than in the case of a hysteroscopy operation. In our standardized protocol we propose that the angle difference should remain within 3 degrees. Furthermore, we recommend that the camera should be color corrected. When the protocol is followed, we show that there are no significant differences between texture features extracted from the same type of tissue (normal or abnormal), but under different viewing conditions. On the other hand, even for the same type of tissue, significant differences arise from large variations in the viewing conditions that do not conform to the protocol (as shown in [18]). More importantly, after applying the proposed protocol, a large number of texture features show significant differences between ROIs extracted from normal versus abnormal tissues. Preliminary findings of this work were published in [18-21].

To the best of our knowledge, although there are guidelines for performing the endoscopy examination, there are no guidelines for the quantitatively interpretation of the results [34,35]. Standardization efforts for reporting endoscopy examinations have been proposed [36]. In this study, we propose a standardised protocol for the analysis of endoscopy images in gynaecological tissue. Following gamma color correction, it was shown that there was no significant difference when investigating experimental tissue in panoramic vs close up views or between two consecutive angles. Most importantly, it was shown that several texture features exhibit significant value differences between normal and abnormal ROIs, for the endometrium allowing the standardized protocol to be employed in Computer Aided Diagnosis systems.

### Recording of endoscopic video

Recent efforts are focused on producing guidelines for gynaecological endoscopy such as gynaecological endoscopy and hysteroscopy [35]. These efforts will help the gynaecologist in standardizing the procedure for capturing endoscopic video and will enable the quantitative analysis of tissue pathology. Similar efforts exist in other endoscopic procedures such as gastrointestinal endoscopy and colonoscopy [34]. Quantitative analysis in these areas is still under investigation. In this study, a complete framework for capturing and analyzing gynaecological endoscopic video is proposed.

### Color correction algorithm

Although the importance of the gamma color correction algorithm is widely recommended in the literature, it has been rarely used. In [9], the authors implemented the color correction algorithm in endoscopic hardware, whereas in [5], the authors implemented color correction in content based retrieval of endoscopic images. In this study, it is recommended that the gamma color correction algorithm is used routinely for correcting endoscopic images. This will facilitate the standardised analysis of endoscopic images.

### Image analysis from experimental tissue for different viewing conditions

It is shown that there was no significant difference in the texture features for panoramic vs close up views and for small consecutive angles in experimental tissue. Gray scale median, variance and entropy were higher in the close up view compared to the panoramic view, whereas contrast and homogeneity were essentially the same in both views. When comparing two consecutive angles, variance was higher in the smaller angle, whereas median, entropy, contrast and homogeneity were in the same range.

In this study, the close up and panoramic view distances were 3 cm and 5 cm respectively. Another study was carried out by our group where the conditions similar to laparoscopy examination were investigated. In that study the close up and panoramic view distances were 4 cm and 7 cm respectively and similar results to this study were obtained [18]. Similar results were also obtained for texture features obtained from different angles (with a difference of 2 degrees).

However, when the distance between the close up vs panoramic views was higher than 6 cm, significant differences in some texture features were obtained. We have also found that some texture feature values exhibited significant differences when the angle differences were more than 5 degrees.

### Multiscale analysis

For completeness, we also report on the results of multiscale analysis. Here, we only report on texture features extracted from the lowpass scales after downsampling the ROIs by 2 × 2 to 10 × 10. We have reported on earlier findings of our group in [18,19]. In multiscale analysis, the physician noted that after downsampling by 4 × 4 to 10 × 10, the ROI images were dramatically altered and offered no basis for diagnosis by visual inspection. From the statistical analysis we have seen a slight variation as a function of the downsampling ratio.

### Human images from the endometrium

We have found that a standardized protocol is necessary in order to eliminate any significant differences that may arise due to the lack of color correction. When the proposed standardized protocol is applied, significant differences in texture features are only due to the desired difference between normal versus abnormal tissue. The standardized protocol is essential for subsequent use of texture features in a CAD system in gynaecological cancer. The protocol is also expected to contribute to increased accuracy in difficult cases of gynaecological cancer.

We hope that the proposed standardized protocol will serve as a starting point for allowing comparisons between different medical centers and images acquired using different medical equipment. In order for this to happen, we require that close-up and panoramic views should differ by about 2 cm. Our findings showed that at a close-up distance of 3 cm and a panoramic distance of 5 cm, there were no significant differences in the texture feature values. Yet, from our earlier findings, a difference of 6 cm yielded significant differences. Similarly, angle differences of the order of 2 to 3 degrees showed no significant differences in the extracted texture features, while an angle difference of 5 degrees yielded unacceptable, significant differences.

Table 10 tabulates the texture characteristics of normal vs abnormal ROIs as these were obtained by interpretation of the texture features values given in Tables 8 and 9.

**Table 10:** Texture characteristics of normal vs abnormal ROIs of the endometrium as these were obtained by interpretation of the texture features values given in Tables 8 and 9.

	**Normal**	**Abnormal**
Gray level	High	Slighthly darker
Variance	Low	Very High
Contrast	Low	High
Homogeneity	Normal range	Slighthly lower
Entropy	Normal range	Slighthly higher

### Concluding remarks

The use of a standardised protocol for capturing and analyzing endoscopic video will facilitate the wide spread use of quantitative analysis as well as the use of CAD systems in gynaecological endoscopy. The proposed standardized protocol suggests the use of color correction and the use of specific viewing conditions so that there will be no significant differences in texture feature values extracted from the same type of tissue (normal or abnormal). On the other hand, when either color correction is not applied or the standardized viewing conditions are not used, significant differences in texture features can arise, even when they come from the same type of tissue. This implies that the proposed standardized protocol cannot be further simplified by reducing any of its requirements. Furthermore, when the proposed protocol is applied, we have found that several texture features can be used to discriminate between normal and abnormal tissue since they exhibit significant differences for the two types of tissue.

Future work will focus on investigating the usefulness of the proposed methodology in other gynaecological clinics, as well as in comparing the findings between the different clinics. Also, a CAD system based on texture features and neural networks is currently under development for classifying between normal and abnormal endometria [21].

Finally, we hope that the proposed system can also be applied to other endoscopic modalities such as colonoscopy and gastroscopy.

## Appendix

### A. Gamma Algorithm

Table 11 gives some of the R, G, and B values for selected testing patterns. Regions of Interest (ROIs) of 64 × 64 pixels were segmented for all colors except for black. The captured images and the digitally generated ones were used for computing the parameters of the gamma correction.

**Table 11:** R, G, and B values for selected target images

**Color**	**R**	**G**	**B**
Black	0	0	0
White	255	255	255
Red	203	0	0
Green	64	173	38
Blue	0	0	142
Dark skin	94	28	13
Light skin	241	149	108
Blue sky	97	119	171
Foliage	90	103	39
Blue flower	164	131	196
Orange	255	116	21
Magenta	207	3	124

**Figure 11:**
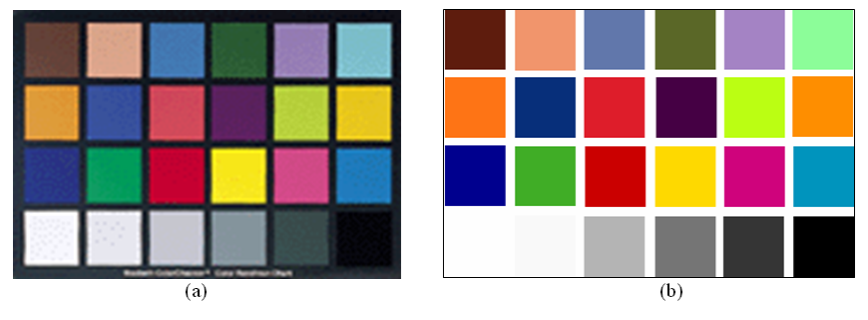
The testing targets of: (a) a color pallet from the Edmund Industrial Optics Company with known color distribution, and (b) the corresponding digitally generated color blocks.

Figure 11 shows the color palette from the Edmund Optics Company [25]. There are 24 colors with known values (Table 11) from which we use all of them except for the black color (R = G = B = 0).

## Authors' contributions

MSN, VT conceived the study, participated in the design of the study, carried out the studies, performed the image processing analysis and drafted the manuscript. CSP and MSP assisted in data analysis and interpretation, and revised the manuscript. ECK and DDK conceived the study, participated in its design and coordination, assisted and revised the manuscript. All authors read and approved the final manuscript.
